# General data protection regulations (2018) and clinical research: perspectives of patients and doctors in an Irish university teaching hospital

**DOI:** 10.1007/s11845-021-02789-8

**Published:** 2021-09-30

**Authors:** Matthew G. Davey, John P.M. O’Donnell, Elizabeth Maher, Cliona McMenamin, Peter F. McAnena, Michael J. Kerin, Nicola Miller, Aoife J. Lowery

**Affiliations:** The Lambe Institute for Translational Research, National University of Ireland, Galway, H91YR71 Ireland

**Keywords:** Data protection, GDPR, Research

## Abstract

**Background:**

Europe’s General Data Protection Regulation, or GDPR, is a set of data protection rules on the acquisition, storage, use, and access of personal data. GDPR came into effect in May 2018 when it was introduced across all 27 European Union (EU) member states and the European Economic Area (EEA). Maintaining compliance with this legislation has presented significant new challenges for ongoing clinical research.

**Aims:**

To evaluate the knowledge and expectations of patients and doctors regarding GDPR and implications for future clinical research.

**Methods:**

An anonymous 12-item questionnaire was circulated to patients and doctors at a University Teaching Hospital. Data analysis included descriptive statistics.

**Results:**

Five hundred nine participants were included: 261 females (51.3%) and 248 males (48.7%). Three hundred fifty were patients (68.8%) and 159 were doctors (31.2%). Three hundred thirty-four participants were aware of GDPR (65.7%): 116 doctors (73.0%) and 218 patients (62.3%, *P* = 0.018). 71.1% of doctors were willing to allow their personal data to be processed anonymously as part of a clinical research project compared to 43.4% of patients (*P* < 0.001). 80.2% of patients believed explicit consent is needed before using personal data in clinical research in comparison to 60.4% of doctors (*P* < 0.001). Level of education impacted awareness of GDPR (*P* < 0.001); a higher level of education among patients increased GDPR familiarity (*P* < 0.001), however failed to impact doctor familiarity (*P* = 0.117).

**Conclusion:**

GDPR has introduced complexity to the processing and sharing of personal data among researchers. This study has identified differences in the perception of GDPR and willingness to consent to data being used in clinical research between doctors and patients. Measures to adequately inform prospective research participants on data processing and the evolving landscape of data protection regulation should be prioritised.

## Introduction

On the 25 May 2018, legislation regarding General Data Protection Regulations (GDPR) was introduced by the European Union (EU), replacing previous data protection laws across Europe [1]. Implementation of these laws was carried out over a staggered 2-year transitional phase across the 27 member states. Initial aspirations for the implementation of the GDPR mandate were to assure European citizens optimal privacy and protection of their personal data, as well as to minimise data breaches which pose a constant threat to personal privacy in today’s data-driven world with many people routinely sharing personal information freely online [2]. GDPR proposed a framework for safeguarding confidential personal information in response to the increasing dissemination of information and the exponential increase in methods of accessing such data. GDPR covers ‘any information relating to an identified or identifiable natural person (‘data subject’)’ (i.e. names, surnames, home address, email address, or an identifier number or data held by a hospital/doctor that could be used to identify a living individual). Additionally, ‘sensitive’ personal data, described in Article 9[1] GDPR include data pertaining to ethnicity, sexual orientation, religious beliefs, trade union membership, and genetic data (chromosomal/DNA) derived from biological samples. The strict interpretation of some of these new legislative changes (in particular, the addition of the Health Research Regulations (HRR) in the Republic of Ireland (ROI)) has the potential to impede clinical research by creating new barriers to overcome in terms of patient consent as well as a lack of clarity in relation to the capacity of international collaborators to share research data [3, 4].

In clinical research, data relating to patient-specific demographics and clinicopathological, therapeutic, and disease-related outcomes are fundamental to robust investigation of epidemiology, disease patterns, and response to existing and novel therapeutic strategies. While the use of personal data is central to all research endeavours, for academic clinicians and medical scientists, the use of identifiable, patient-specific data is indispensable for research which requires long-term follow-up of patient outcomes from a specific disease process or following a particular treatment/intervention [5–7]. There is concern among the clinical research community that the most recent data protection regulations may provide inherent obstacles for stakeholders involved in performing clinical research in Ireland. These include the mandatory requirements for project-specific ‘explicit consent’ for each data subject (or participant) before inclusion in research, the reconsenting participants for inclusion in studies using biobank/archived material, or for inclusion in retrospective studies [4, 8, 9]. The efforts of the HRR coincide with GDPR in relation to health-related research, outlining suitable and specific safeguards required when processing personal data for health research in the ROI [4]. It is anticipated that these new regulations will reduce data breaches; however, the full implications of the new restrictions are yet to be determined; under current regulations, the right to erasure (Article 17.3) directly conflicts with and impedes the principles of clinical research [10, 11]. Additionally, the mandatory requirement to obtain further consent should the research project aims change removes the flexibility that is so crucial to performing dynamic and relevant clinical research. Furthermore, increasing restrictions regarding data sharing are likely to render international collaborative projects increasingly more difficult to establish and run, which may lead to clinicians relying on smaller patient data from single centres, reducing the robustness of results obtained.

Although HRRs are explicit, the views and perspectives of clinicians and patients in relation to GPDR and its potential implications for clinical research are less certain. The aim of this study was to evaluate the views and perspectives of patients and doctors as to how personal data is processed following the introduction of GDPR and their preferences in this regards as well as the potential implications of its introduction for future clinical research.

## Methods

**Fig. 1:**
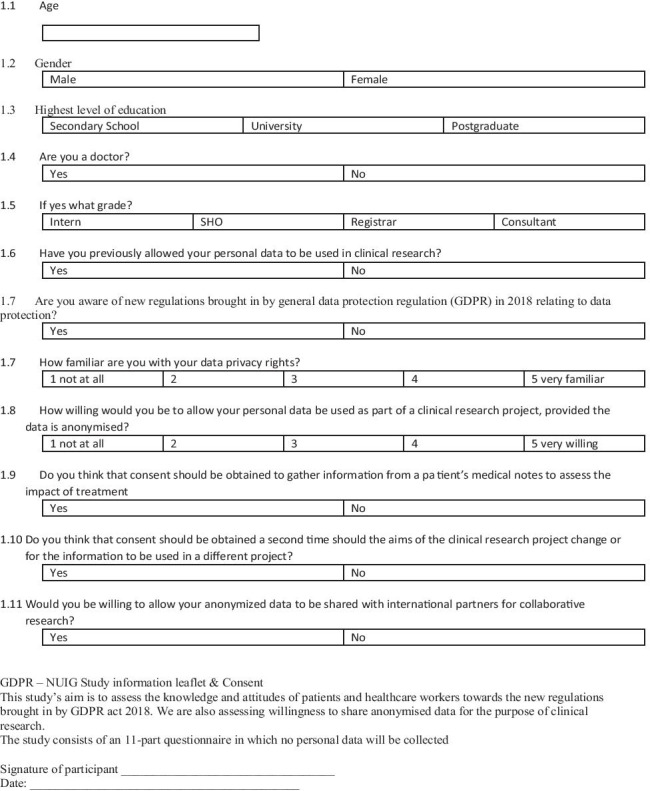
Questionnaire distributed to participants in this study

Local ethical hospital approval was obtained from the Clinical Research Ethics Committee (CREC) at Galway University Hospital (GUH). A 12-item questionnaire was designed with respect to demographic, research experience, and prospective of GDPR, as well as questioning pertaining specifically to consent (Fig. 1). At present, there are no validated questionnaires for the investigation of this topic in existence. Each participant was asked to participate in the study having been addressed by one of the four independent data processors (EM, CM, MGD, and JPOD) during October 2019–September 2020. Doctors working in GUH were invited to partake in the study while at work in the hospital. Patients attending medical and surgical outpatient clinics were also invited to partake in the study at the time of their hospital visit. Each participant conducted the survey in writing, with no external questioning from researchers. No participants were recruited to undertake the study through electronic surveys, and no incentive was offered to any participants for their participation in the survey. The Irish National Framework of Qualifications (NFQ) was used to classify levels of education [12].

Data was stored on a password protected file on a password protected computer in the partner academic institution. All data retrieved was anonymised. Data protection was in full compliance with EU GDPR guidelines (2018). Data analysis was performed using Statistical Package for the Social Sciences (SPSS) version 26.0. Fisher’s exact (†), Chi-squared (χ^2^), and one-way analysis of variance (ANOVA, ‡) tests were used as appropriate.

## Results

### Demographics

There were 509 participants included in this study; of these, 261 were female (51.3%) and 248 were male (48.7%). There were 350 patients (68.8%) and 159 doctors (31.2%) participating in this study. Details of clinical, educational, and previous research characteristics of participants are outlined in Table 1. Of the doctors included in the study, 108 were interns (67.9%), 33 were senior house officers (20.8%), 14 were registrars (8.8%), and 4 were consultants (2.5%).

**Table 1:** Clinical, educational, and research characteristics of participants in this study

**Characteristic**	**Overall cohort (*N* = 509)**	**Patients (*N* = 350)**	**Doctors (*N* = 159)**	***P*-value**
**Gender** **Female** **Male**	261 (51.3%) 248 (48.7%)	160 (45.8%) 190 (54.2%)	88 (55.3%) 71 (44.7%)	0.095 †
**Mean age (± SD, mean, range)**	49.9 years (± 21.6, 48, 19–92)	60.2 years (± 17.7, 62, 19–92)	26.9 years (± 6.6, 25, 22–64	< 0.001* ‡
**Levels of education** **Secondary** **Third level** **NFQ level 9** **NFQ level 10**	78 (15.3%) 239 (47.0%) 166 (32.6%) 26 (5.1%)	78 (22.3%) 165 (47.1%) 95 (27.1%) 12 (3.4%)	0 (0.0%) 74 (46.5%) 71 (44.7%) 14 (8.8%)	< 0.001* χ^2^
**Previous allowed data in clinical research** **Yes** **No**	159 (31.2%) 350 (68.8%)	77 (22.0%) 273 (78.0%)	82 (51.6%) 77 (48.4%)	< 0.001* †
**Aware of GDPR** **Yes** **No**	334 (65.6%) 175 (34.4%)	218 (62.3%) 132 (37.7%)	116 (73.0%) 43 (27.0%)	0.018* †

*SD* standard deviation, *GDPR* General Data Protection Regulations, † Fishers’ exact test, ‡ one-way analysis of variance, χ^2^ Chi-squared test.

^*^Denotes statistical significance.

### Subgroup analysis: participants and GDPR

**Fig. 2:**
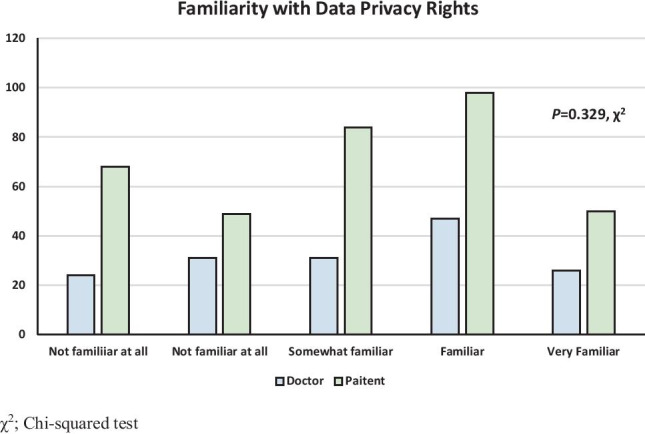
Patient and doctor familiarity with their data privacy rights

**Fig. 3:**
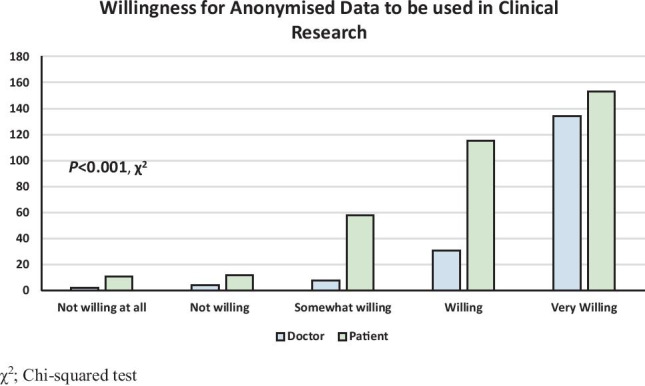
Patient and doctor willingness for with their data to be used

Overall, 159 participants had previously consented to allow their data to be used in clinical research (31.2%, 159/509). Of these, doctors were more likely to have agreed for their data to be used in clinical research (51.6% *vs.* 22.0%, *P* < 0.001 †). Overall, 334 participants (65.7%) were aware of GDPR implementation, and doctors were more likely to have awareness (73.0%, vs. 62.3%, *P* = 0.018 †). There was no difference between in the degree of familiarity with the more granular/specific details of data privacy rights between doctors and patients (*P* = 0.329 χ^2^) (Fig. 2). Doctors were significantly more willing to allow their personal data be used as part of a clinical research project, provided the data is anonymised (71.1% vs. 43.4%, *P* < 0.001 χ^2^) (Fig. 3).

### Subgroup analysis: clinical research

Doctors were significantly more likely to believe explicit consent should be obtained to gather information from a patient’s medical notes and used in clinical research projects (80.2% vs. 60.4%, *P* < 0.001 †). Both cohorts were as likely to believe consent should not be obtained a second time, should the aims of the clinical research project change or for the information to be used in a different project (52.6% vs. 44.0%, *P* = 0.152 †). Doctors were significantly more likely to allow their data to be used in an international research collaboration (91.8% vs. 72.6%, *P* < 0.001 †). When assessing patients and doctors independently, being involved in previous research projects impacted patient awareness of GDPR (*P* = 0.031 †), however did not impact doctors awareness of GDPR (*P* = 0.286 †). Being involved in previous research was positively associated with both patient and doctors willingness to become involved in future research projects (*P* = 0.002 and *P* = 0.027 respectively, both †).

### Subgroup analysis: education level

Level of education impacted awareness of GDPR (*P* < 0.001, χ^2^); both patients and doctors with a higher level of education were more likely to be aware of GDPR (*P* < 0.001 and *P* = 0.027, both χ^2^). Higher level of education among patients increased their familiarity with GDPR (*P* < 0.001, χ^2^), however failed to impact doctor familiarity with the regulations (*P* = 0.117, χ^2^). The level of education impacted the willingness of participants to allow for their personal anonymised data be used as part of a clinical research (*P* < 0.001, χ^2^). Doctors with higher levels of education were willing to provide their anonymised data in clinical research (*P* = 0.014 χ^2^), however level of education did not alter patient willingness to do so (*P* = 0.219, χ^2^). Higher level of education among patients failed to increase their willingness have their data shared in an international research collaboration (*P* = 0.358, χ^2^), however did impact doctors willingness (*P* < 0.001, χ^2^).

## Discussion

The current study demonstrated the views and perspectives of patients and doctors towards the newly implemented data protection regulations. The level of education received by participants seems to have impacted patient’s awareness of new data protection regulations and familiarity with the details of GDPR, as well as patients’ willingness to contribute their anonymised data towards international research collaborations. Doctors were slightly better informed than patients in relation to the importance of GDPR in clinical practice; however, 27% of doctors admitted to being unaware of the regulations, despite the regulations being implemented into practice by the European Union since May 2018 for citizens of the EU. Since then, academic and medical institutions have been expected to comply with regulations when processing patient data for clinical and research purposes. These prove to be worrying findings; initial aspirations for GDPR were ‘to provide rules for the protection of the personal data of natural persons and the processing of their personal data’, and the current study highlights only a moderate awareness of GDPR among both patients (63%) and doctors (73%). With this in mind, the anticipated obstruction to clinical research seems to be of secondary importance as it remains crucial that doctors and patients are careful to protect data in the clinical setting, in order to prevent data breaches [4, 8, 9, 13].

In this analysis, 27% of doctors and 40% of patients reported being unaware of GDPR legislation, with both illustrating comparable familiarity with data protection rights. This represents some concerning findings; doctors are expected to process large volumes of sensitive patient data on a daily basis, where protection of healthcare data is of the greatest importance. Furthermore, there has been a vogue in recent times to educate healthcare staff of the importance of data protection [14], with Ireland’s health service executive (HSE) attempting to educate their staff of GPDR and its implications for their clinical work [15]. Thus, it is surprising that a large proportion of doctors admit unawareness in relation to data protection. However, acknowledgement for several other confounding factors which impact awareness of GDPR must be accounted for: Markopoulou et al. highlighted level of education to impact upon awareness of GDPR [16], which is replicated by data in the current study, with levels of education correlating with the both patient and doctor’s awareness and understanding of the significance of GDPR. Furthermore, in our patients, the level of education received impacted awareness of GDPR (*P* < 0.001), which brings into question the delivery of information to our patients with respect to issues such as data protection, as well as in relation to the day-to-day discussions on topics such as disease diagnoses, prognoses, and management. O’Sullivan et al. performed a quantitative analysis illustrating the complexity of clinical research patient information leaflets/informed consent forms when compared to traditional readability criteria [17] and health literacy-based tools [18], which somewhat explains the difficulties people have in understanding the importance of complex issues, such as data protection. GDPR Article 5 indicates that personal data should be processed in a lawful, fair, and transparent manner [3], which highlights that efforts must be made to ensure concise and coherent education of patients in language which is deemed appropriate for the recipients to understand, without overcomplicating delivery unnecessarily.

While 40% of patients admit a deficit in awareness of GDPR, further data from the current analysis suggests 27% doctors are unaware of new data protection regulations. This is extremely concerning: Efforts from governing bodies such as the Royal College of Physicians of Ireland, Royal College of Surgeons in Ireland, and the Irish College of General Practitioners have made efforts to educate doctors of the significance of GDPR for their clinical practice [19–21], as have the HSE through mandatory online learning modules for staff [15]. Moreover, the *Irish Medical Journal* has attempted to educate readership through editorial commentary regarding GDPR and its implications for clinical practice [22]. Despite these efforts, a 2020 study from Wallace et al. reported that almost 90% of Irish non-consultant hospital doctors (NCHDs) believe specific training in the fields of GDPR and Irish research regulations would be useful, although only 25% described having received such training [23]. This is surprising as the HSE has implemented online training modules which are specific to GDPR implementation and expected practice [15]. In Wallace’s study, doctors suggested a lack of educational resources, information, and training opportunities for clinicians in relation to data protection. Data from Wallace et al. indicate that 89% of clinicians are keen for involvement in clinical research, while 87% believe the participation of doctors in clinical research being ‘important.’ In spite of this, results from the present study suggest only 1/6th of doctors believe they are ‘very familiar’ with GDPR regulations; this highlights the potential for significant data breaches in the setting of clinical research, and the fundamental requirement for an in-depth understanding of GDPR for any stakeholder in the clinical research [4]. Furthermore, a study from Corbett et al. illustrated the requirement for junior doctors working in an Irish hospital to receive education in relation to electronic data protection in healthcare [24]. Thus, novel strategies educating both doctors and patients may be warranted, or making a conscious effort in clinical areas to provide HSE data privacy statements in clinical areas to ensure transparent processing of patient data in our hospitals. These efforts may be successful in heightening the appreciation for GDPR implementation in our workplace, in order to counteract potential preventable data breaches in future.

In the current study, approximately 60% of doctors and 80% of patients believed explicit informed consent to be an essential component in the recruitment of participants into clinical research studies. The informed consent process is a critical cornerstone of the ethics underpinning medical treatments, surgical procedures, and participation in clinical research [25]. Prior to May 2018 in Ireland, a data subject was not required to provide explicit consent for inclusion in a clinical research study, and data processing was permitted using consent naïve data. Since the implementation of GDPR, Irish legislation via the HRR requires data minimisation, pseudo-anonymisation, and anonymisation where possible, and explicit consent from the data subject being a mandatory prior to inclusion in clinical research [1, 2, 4]. This differs from other European states where explicit consent is not a requirement, as set out in Article (89) 1. Although the ROI has introduced less lenient (or more ‘obstructive’) regulations than other member states, 281 of the 350 included patients believe this to be appropriate, reiterating positive aspects to strict GDPR implementation. Debate in recent times suggests modification, or indeed reformation, of the current informed consent process for clinical research, and deliberation of such changes is underway as we attempt to compete with the ever-evolving challenges in an environment becoming increasingly exposed to medicolegal hazards [26, 27]. On the contrary, over 50% of doctors and patients included in this study believed that a second formal consent process is unnecessary to use personal data for another project. This projects the potentially ‘disruptive’ nature of GDPR in the setting of biobanking, cross-border data transfer, and databanking, all processes which have been restricted since the implementation of GDPR [28]. The sequelae of this are yet to be observed; however, a forecasted reduction in the volume and impact of clinical research is possible. Thus, the requirement to obtain further consent, should the aims of the project change from the initial aspirations, represents a significant practical barrier to research brought in by EU’s GPDR 2018.

Three-quarters of patients and over 90% doctors were willing to allow their clinical data to be used in international research collaborations. The amalgamation of large volumes of pooled data is central to delivering the highest quality scientific evidence to enhance diagnostics, guide therapeutic decision making, and inform patient prognosis. Fostering international collaborations between research institutions is vital to allow large prospective research projects to progress. The strict interpretation of this legislation may be questioned as to what benefit it ultimately provided to patients while representing a potentially unnecessary obstacle to research.

The study suffers from being conformed from a single-centre questionnaire, including only 12 data points, which limits the conclusions which can be drawn from this analysis. Within this study, we evaluated the two very different participating subgroups (patient mean age 60.2 years vs. 26.9 years for doctors) with varying levels of education received. However, in spite of this, we present real-world views and perceptions of patients and doctors regarding GDPR, using the first ever GDPR questionnaire.

The implementation of the EU GDPR and the Irish HRR has introduced additional complexity relating to the processing and sharing of data among researchers. This study has identified differences in the perception of GDPR and willingness to consent to data being used in clinical research between doctors and patients. Measures to adequately inform prospective research participants on data processing and the evolving landscape of data protection regulation should be prioritised, with emphasis upon the conscious processing of patient data in a transparent manner, with the formal implementation of GDPR education strategies to envisage how clinical research may be carried out in a safe and GDPR compliant manner in the future.
